# Reply to: “Comment on volcanic monitoring of the 2021 La Palma eruption using long-period magnetotelluric data”

**DOI:** 10.1038/s41598-025-30554-9

**Published:** 2025-12-09

**Authors:** P. Piña-Varas, J. Ledo, P. Queralt, DMartínez van Dorth, A. Marcuello, I. Cabrera-Pérez, L. D’Auria, A. Martí

**Affiliations:** 1 Departament de Dinàmica de La Terra I de L’Oceà, Facultat de Ciències de La Terra. Universitat de Barcelona, Barcelona, Spain; 2 Departamento de Física de La Tierra y Astrofísica, Facultad de Física, Universidad Complutense de Madrid, Madrid, Spain; 3 Instituto Volcanológico de Canarias (INVOLCAN), Canary Islands, 38600 Granadilla de Abona, Tenerife, Spain

**Keywords:** Geophysics, Applied physics

Replying to: D. Cordell et al; Scientific Reports 10.1038/s41598-025-30551-y (2025).

As described in C_DC24, the main concern of the authors is related to the quality of the N-S electric field measurements. They propose two possible explanations for the observed XY and XX impedance tensor components reported: (1) problematic electric field data on the N-S direction and/or (2) near-surface distortions in the north–south geoelectric field measurement. We will therefore address both issues separately.

## Data reliability

In the first part of C_DC24 the authors argue that the resistivity variations shown in PV23 are partly due to instrumentation problems. This is primarily due to the fact that the observed resistivity changes do not affect all impedance tensor components, but only the XY and XX components show noticeable changes with time.

The main explanation proposed in C_DC24 for this behaviour is a problem with the measurement of the north–south geoelectric field dipole, related to electrodes instabilities or rainfall events.

Our time series record begins when the volcanic eruption is still active (5/11/2021), and ends approximately one year after the volcanic eruption ceased (22/01/2023). There is a time hiatus between 26/07/2022 and 24/11/2022 due to a damaged electrode. C_DC24 authors propose precipitation events on the eastern part of the island as a potential cause of the N-S electric field instabilities although the LMT station is located on the western flank and there are important climate contrast between both slopes of the island^2^. During the time period analysed in PV23, there were several precipitation events in La Palma Island^3^, and no significant correlation between precipitation events and electric field behaviour could be inferred (See figures and raw data in the following link: 10.34810/data1071).

**Fig. 1:**
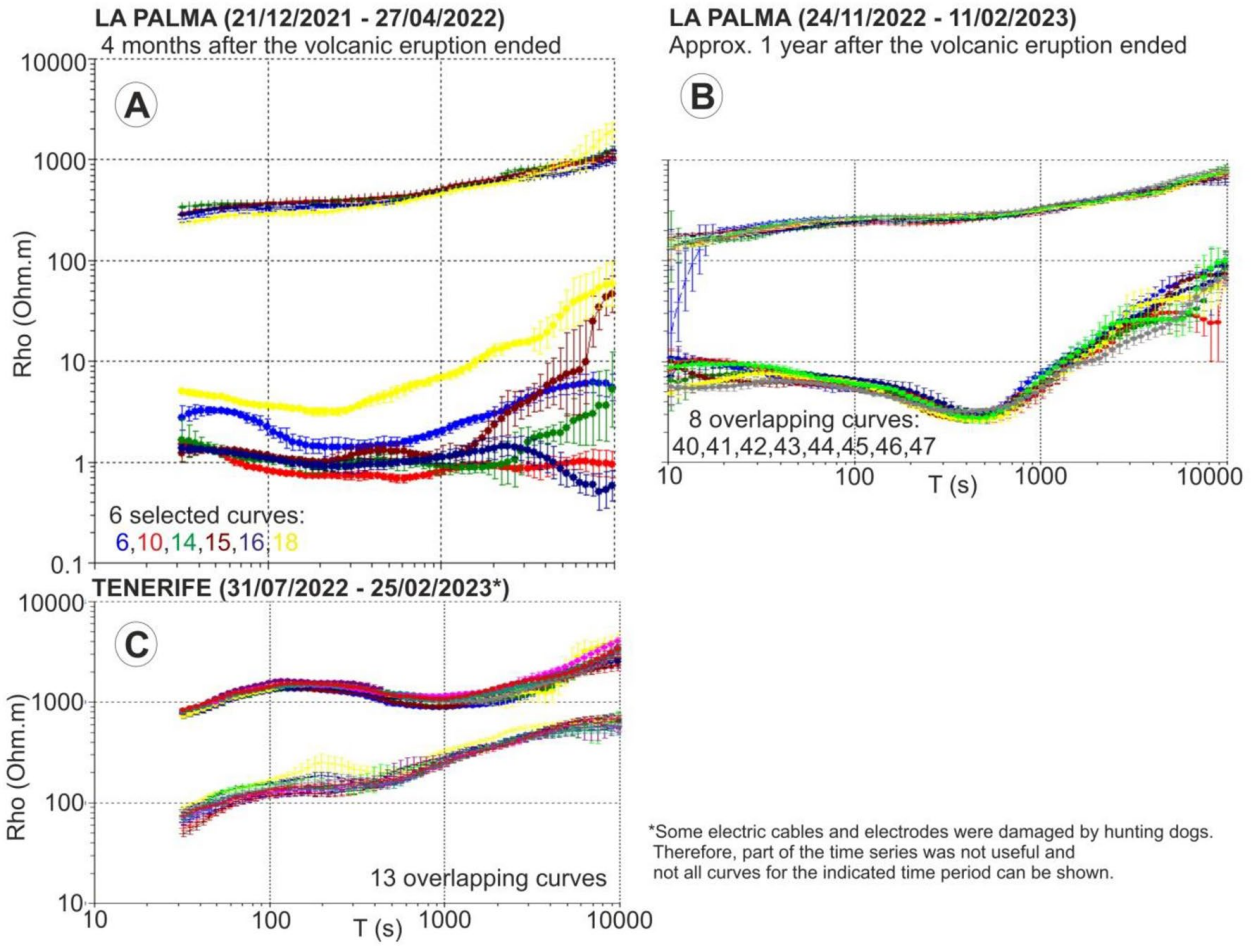
Off-diagonal apparent resistivity curves for different temporal MT impedances obtained by processing the original time series every ten days. (**A**) Data from La Palma MT site during the first four months after the volcanic eruption. (**B**) Data from La Palma MT site approximately one year after the volcanic eruption. (**C**) Data from an MT site located on the island of Tenerife, as an example of other long-period data acquired and processed following the same methodology.

Most of the reported rainfall events are less than 5–6 mm, which are typically considered light rain, and only one event with 32 mm was reported at the Tazacorte weather station^3^ on 26/03/2022. This rainfall occurred in the 10 days corresponding to the MT curve labelled 15 in Fig. 1A. Figure 1A shows six selected PV23 curves in which significant resistivity changes with time are observed. Curve 15 is plotted in dark red, while the MT curves corresponding to the 10 days before and after (curves 14 and 16) are plotted in green and dark blue, respectively. As shown in the figure, although the largest rainfall episode has been recorded during these 30 days, the largest changes in electrical resistivity are not observed at this time period.

Therefore, we do not consider that rainfall is causing the observed changes in resistivity. Moreover, we find it particularly unlikely that precipitations over an area of 100 m by 100 m would only affect the N-S direction. If the rainfall is causing electrodes instability, both N-S and E-W components should be affected. C_DC24 also suggest that variations in the shallow hydrologic structure near the measurement site may be the cause of the observations, however the water level heads during the eruption shows a groundwater level variation in the closest piezometer (LP-07) to our LMT site of less of 0.5 m during the eruption period^4^.

As for other possible causes mentioned in C_DC24, we do not consider that it is due to instabilities of the electrodes, but rather we believe that it has a geological origin and is largely related to processes linked to the volcanic activity itself (including the post-eruptive phase).

The non-polarizable Pb-PbCl_2_ electrodes employed in this installation were newly manufactured and deployed following the standardized protocol established through the research group’s extensive experience in magnetotelluric (MT) surveys. The installation procedure adhered to established best practices: electrodes were buried at maximum feasible depths given site-specific constraints, bentonite was applied at the base to optimize electrical contact, contact resistance measurements were recorded, etc.

Contact resistance measurements for both electrical components yielded values in the range of 10–25 kΩ, which were considered elevated but acceptable. These resistance values were considered acceptable given the specific soil characteristics and geological conditions present at the monitoring site.

During the scheduled maintenance visits, conducted approximately every three months, a comprehensive series of routine tasks were systematically performed. These procedures included verifying battery condition and charge regulator functionality, cleaning the solar panel, downloading recorded data, confirming the stability of magnetometer orientation and levelling, inspecting electrical cables for potential animal-induced damage (such as biting or cutting), and measuring contact resistances.

With respect to the contact resistance measurements, the recorded values consistently remained within the previously established ranges until the maintenance visit conducted in November 2022, when malfunctions were identified in the NS electrical component, specifically, one of the electrodes ceased functioning properly. Consequently, as detailed above, a data gap exists in the reported results spanning from 26 July 2022 to 24 November 2022.

New electrodes were installed and the electrical dipole configuration was enhanced through duplication. Given that LEMI systems are equipped with four electrical channels; this modification enabled each electrical component (Ex and Ey) to be measured using two independent electrical dipoles. This redundant configuration was implemented to mitigate the risk of future data loss in the event of single electrode failure, with the secondary dipole serving as a backup system.

It is important to note that both the primary resistivity variations observed (LP1 to LP24 in Fig. SM3) and the initial stabilization of the magnetotelluric responses (LP25 to LP27 in Fig. SM3), which occurred prior to 26 July 2022, were recorded using the original electrode configuration. This temporal sequence ensures data consistency across these critical measurement periods and confirms that the observed phenomena were captured.

To rule out technical problems associated to the site installation Fig. 1B shows eight curves corresponding to data recorded during two and a half months at the exact same MT site used in PV23 but 12 months later (contact resistances of the same order of magnitude as before). In this case, no significant changes in electrical resistivity with time are observed.

Figure 1C shows the results obtained after recording for more than four months at a MT site located on the island of Tenerife, which has some geographic and geological similarities with La Palma. No significant changes in electrical resistivity over time are observed in Tenerife, where the same methodology was applied: same type of datalogger, same type of electrodes (contact resistances in the range of 5 kΩ), same technicians in charge of the installation, same processing workflow, etc.

All this rules out, therefore, that acquisition problems or instabilities in the measurements related to the meteorological characteristics of the island are the main cause of the observed temporal resistivity changes.

Finally, C_DC24 mentions the fact mention the fact that data showing out-of-quadrant phases (POQ) “may also point to a deeper underlying issue with the data”. We do not quite understand what the authors mean by “deeper underlying issue”. However, if their statement suggests that the anomalous phases are attributable to measurement-related issues, we must disagree with this interpretation based on the available scientific evidence. POQ have been extensively documented as a common phenomenon in magnetotelluric measurements, particularly in regions characterized by complex geological settings and intricate three-dimensional subsurface structures (e.g.,^5–10^). This anomalous behavior has been consistently attributed in the literature to significant variations in electric field direction (e.g.,^11–13^), reflecting the inherent complexity of electromagnetic field interactions in heterogeneous media. Importantly, our review of the existing literature reveals no established correlation between POQ occurrences and data acquisition deficiencies or instrumentation malfunctions, including electrode-related problems.

## Modelling and interpretation

In the second part of C_DC24 the authors propose that: “Our hypothesis is that the large variations in the MT data are due to electrode instabilities in the north–south geoelectric field measurement, rather than deeper (> 1 km) variations in the magmatic system. If this hypothesis is correct, we might expect these issues with the geoelectric field measurement to manifest as near-surface artefacts in the inversion model near the MT measurement site.” To justify this affirmation they carried out a few forward modelling computations using simple 3-D models.

**Fig. 2:**
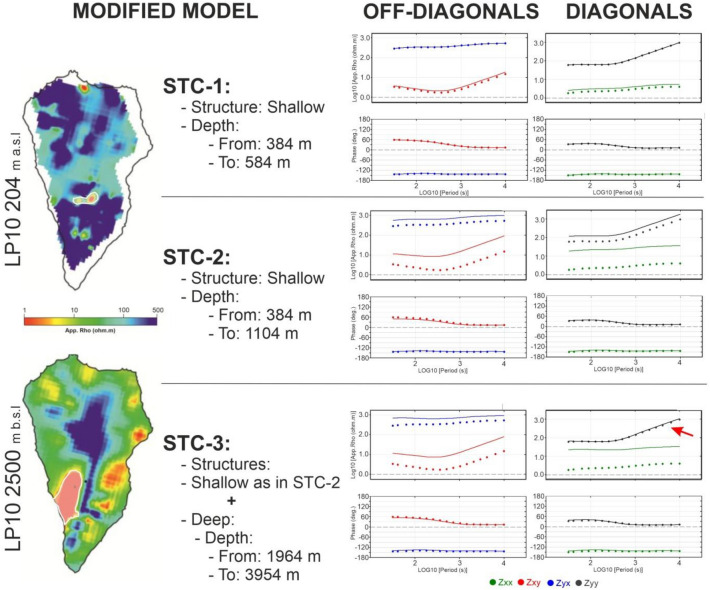
Sensitivity tests performed against shallow and deep structures resolved in the LP10 model. Modified structures are highlighted in white in the left map views of the 3-D inversion model. Upper panel: STC-1 model in which only the shallowest part of the shallow structure has been modified. Central panel: STC-2 model in which the entire shallow structure has been modified. Lower panel: STC-3 model in which both the shallow (as in STC-2) and deep structure have been modified. Dots: MT responses of the LP10 model; Lines: MT responses of the corresponding modified model. Blue: YX, Red: XY, Green: XX; Grey: YY. Red arrow: indicates the YY component in the STC-3 model.

We will not go into the discussion of their synthetic models, but we should mention that we found a couple of inconsistencies between the synthetic models presented in C_DC24 and the idea of testing the structure resolved in PV23 around 200 m a.s.l. First, the orientation of the structure in C_DC24 is N-S, as opposed to the structure solved in PV23, which is roughly E-W. Secondly, and more importantly, the structure resolved in PV23 is located around 1 km depth (~ 200 m a.s.l); while the structure in C_DC24 is much shallower located at 200 m depth (1.07 km a.s.l, Fig. 2 in C_DC24).

The inversion models presented in PV23 demonstrate that the shallowest depth at which a conductive body proximate to the LMT site can be resolved is approximately 400 m. To assess the sensitivity of the magnetotelluric responses to shallow conductivity variations, the conductive zone in model LP10 was replaced with a resistivity value of 40 Ω·m, representing the average resistivity of the corresponding area resolved in model LP6. This modification, designated as Modified model STC-1, produced no discernible impact on the computed MT responses (Fig. 2). These results indicate that conductivity variations occurring solely within the 400 m depth range cannot account for the observed magnetotelluric responses (Fig. 2, upper panel).

In contrast, when the modification was extended to include the deeper portion of the conductive structure (approximately 700 m in thickness), designated as Modified model STC-2, significant variations were observed across all four components of the impedance tensor (Fig. 2, central panel).

A subsequent modification to model STC-2, termed STC-3, incorporated the replacement of the deep north–south trending conductive body located on the western flank. The results reveal that through the combined modification of both shallow and deep western structures, the YY component of the impedance tensor returns to its baseline values. This behavior closely parallels the observed field responses, in which the YX and YY components remain essentially unchanged throughout the monitoring period (Fig. 2, lower panel).

These systematic model modifications provide valuable insights into the depth-dependent sensitivity of magnetotelluric measurements and demonstrate that the observed temporal variations in resistivity responses are primarily controlled by changes in deep structural elements rather than shallow conductivity variations alone.

The sensitivity analyses reveal two critical findings: (1) the observed magnetotelluric variations cannot be attributed to a single shallow subsurface structure, but rather result from the complex interaction of multiple structures distributed across different geological zones and depth intervals; (2) the magnetotelluric data exhibit significant sensitivity to resistivity modifications within these heterogeneous structures, encompassing both shallow and deep components, notwithstanding the constraint of single-site measurements.

The availability of a pre-eruption baseline 3D MT model provided us with a valuable foundation and a unique research opportunity to investigate the spatial variations in resistivity associated with this significant geological event. At the same time, we are aware that having a single MT site the uncertainty on the new 3D model is notable but we think that the main electrical structures are required to fit the data.

Regarding the capabilities of long-period MT measurements for monitoring volcanic activity, the hypothesis argued by the authors is mainly based on the fact that all previous temporal MT studies at volcanoes used broadband data. This was, indeed, one of the main motivations for conducting the La Palma monitoring experiment: to test with real data and during an active volcanic eruption the potential of long-period measurement for this type of studies. In this regard, the long-period data collected on La Palma have proven to be of great interest as they allow detecting temporal changes in resistivity associated with the volcanic activity. We agree that higher frequencies in theory would help to better resolve the crustal inductive effects, but the capability to detect changes in resistivity will depend on whether such changes occur within the skin depth range of the measured frequencies, which will depend considerably on each study area and is very difficult to know in advance. Perhaps, as a standard procedure, an even wider frequency range should be considered and broadband and long-period data should be measured at the same MT site.

In summary, we have addressed the main concerns of the authors by providing evidence to support the value of our work. On the one hand, the explanations provided in this document rule out possible problems during data acquisition or rain-related effects at very shallow depths as the cause of the observed MT responses. On the other hand, the sensitivity tests performed indicate that complex resistivity variations along different structures and depths may be the cause of the observed MT responses, rather than due to a single local near-surface structure.

We appreciate the interest shown in our work and the comments of our colleagues, as this type of debate helps us all to continue to increase our knowledge and improve future volcanic monitoring experiments using the MT method.

## Supplementary Information

Below is the link to the electronic supplementary material.

Supplementary file1
